# Total body irradiation: A transition from a Co‐60 treatment unit to an IMRT lateral‐field extended‐SAD technique

**DOI:** 10.1002/acm2.14430

**Published:** 2024-07-01

**Authors:** Anastasia Kolokotronis, Malik Brunet‐Benkhoucha, Étienne Roussin, Julie St‐Pierre, Eve‐Lyne Marchand, Maryse Bernard

**Affiliations:** ^1^ Département de Radio‐oncologie CIUSSS de l'Est‐de‐L'Île‐de‐Montréal Hôpital Maisonneuve‐Rosemont Montréal Quebec Canada

**Keywords:** extended‐SSD IMRT, TBI commissioning, TBI treatment planning, total body irradiation (TBI)

## Abstract

**Purpose:**

The purpose of this work was to detail our center's experience in transitioning from a Co‐60 treatment technique to an intensity modulated radiation therapy (IMRT) based lateral‐field extended source‐to‐axis distance (e‐SAD) technique for total body irradiation (TBI).

**Materials and Methods:**

An existing beam model in RayStation v.10A was validated for the use of e‐SAD TBI treatments. Data were acquired with an Elekta Synergy linear accelerator (LINAC) at an extended source‐to‐surface distance of 365 cm with an 18 MV beam. Beam model validation measurements included percentage depth dose (PDD), profile data, surface dose, build‐up region and transmission measurements. End‐to‐end testing was carried out using an anthropomorphic phantom. Treatments were performed in a supine position in a whole‐body Vac‐Lok at an e‐SAD of 400 cm with a beam spoiler 10 cm from the couch. Planning was achieved using IMRT, where multi‐leaf collimators were used to modulate the beam and shield the organs at risk. Beam's eye view projection images were used for in‐room patient positioning and in‐vivo dosimetry was performed for every treatment.

**Results:**

The percent difference between the measured and calculated PDD and profiles was less than 2% at all locations. Surface dose was 83.8% of the maximum dose with the beam spoiler at a 10 cm distance from the phantom. The largest percent difference between the treatment planning system (TPS) and measured data within the anthropomorphic phantom was approximately 2%. In‐vivo dosimetry measurements yielded results within the 5% institutional threshold.

**Conclusion:**

In 2022, 17 patients were successfully treated using the new IMRT‐based lateral‐field e‐SAD TBI technique. The resulting clinical plans respected the institutional standard. The commissioning process, as well as the treatment planning and delivery aspects were described in this work with the intention of supporting other clinics in implementing this treatment method.

## 1 INTRODUCTION

Total body irradiation (TBI) continues to be an important part of conditioning regimens for allogeneic hematopoietic stem cell transplantation.^1^ The choice of conditioning regimen is based on different considerations including patient characteristics, diagnostic and associated risk factors.

TBI is a crucial component in myeloablative conditioning regimens. It is used to eradicate malignant cells while providing immunosuppression to prevent rejection of engrafted hematopoietic cells. At our center, patients undergoing myeloablative transplantation are most frequently treated with our institutional standard TBI fractionation of 12 Gy in 6 fractions delivered over 3 days, twice daily with a minimum of 6 h in‐between each fraction.^2^, ^3^, ^4^ Alternative reduced‐intensity conditioning regimens, whether with lower dose or without TBI, are also viable options. For a non‐myeloablative conditioning transplant, TBI of 4 Gy in 2 fractions or 2 Gy in 1 fraction is frequently used.^5^

Previously at our center, Cobalt‐60 (Co‐60) irradiators at extended source‐to‐surface distance (e‐SSD) were used to deliver TBI treatments. However, there are radiation safety concerns associated with housing a radioactive source and administrative burdens in regards to regulations on radioactive devices. Recently, the health ministry of Quebec has encouraged an end to the use of Co‐60 sources in hospital settings for patient treatments. Alternative delivery methods for TBI have been developed over the years and validated. Some such methods which have already been proposed include AP/PA, helical tomotherapy, open lateral fields at e‐SSD, multi‐isocentric VMAT and e‐SSD VMAT.^6^, ^7^, ^8^, ^9^, ^10^ There is no gold standard for TBI and implementing a new clinical technique is challenging, as there are few guidelines on how to choose the best approach for TBI and acquire data for the purpose of commissioning and validating the technique. The last AAPM task group (TG) report was published in 1986, TG‐29, with specifications and general considerations for performing total and half‐body photon irradiation techniques.^10^ The AAPM has initiated a new TG to provide recommendations and guidelines for introducing TBI in a clinical setting, TG‐379.

Long‐term and clinically significant side effects associated with TBI include interstitial pneumonitis and chronic kidney disease, which are linked to the absorbed dose to the lungs and kidneys.^11^ In the literature, efforts to minimize complications often involve keeping the mean lung dose below 8–9 Gy, even though other studies have reported mean lung dose exceeding these thresholds.^2^, ^8^, ^12^, ^13^ Additionally, as a conservative approach, dose‐rates are generally kept below 20 cGy/min.^4^ However, it is still debated whether factors, such as dose‐rate, can have a negative impact on the lungs and/or kidneys and may induce pneumonitis and/or chronic kidney disease.^14^, ^15^, ^16^ While prior studies have correlated increased dose‐rate with toxicity effects to the lungs, a recent VMAT study by Melton et al. showed that increased dose‐rate from 40 to 100 MU/min did not correlate with an increased lung toxicity in their patients’ cohort.^17^

Dose‐rate, mean lung dose, and their correlation are important factors to consider when choosing a treatment technique, as they are still subjects of clinical trials.^1^, ^18^ After careful evaluation of different TBI techniques, we selected an IMRT lateral‐field extended source‐to‐axis distance (SAD) technique at 400 cm with the patient in a supine position in a whole body Vac‐Lok. The chosen dose‐rate was 140 MU/min (8.75 cGy/min), which delivered a similar dose‐rate to the body as our previous Co‐60 based treatments (14.20 cGy/min in 2016 to 6.30 cGy/min in 2022). For this reason, we expected to maintain consistency with historical outcomes and side effects.

The IMRT lateral‐field e‐SAD technique, developed at our center through a multidisciplinary approach, was adapted from the Fog et al. experience and was officially implemented as of June 2022.^8^ The main distinction between the two methods lay in the dosimetry planning stage, where our strategy employed inverse IMRT planning as opposed to manual multi‐leaf collimator (MLC) placement and beam weight adjustment. Regarding clinical objectives, minor variations were observed in the planning aims concerning planning target volume (PTV) coverage but remained consistent for the lung dose criteria. For a prescription of 12 Gy in 6 fractions, Fog et al. reported a minimum dose (*D*_min_) coverage to the PTV of at least 10.8 Gy (*D*_min_ >90%), whereas our minimum PTV coverage was at least 10.4 Gy to 95% of the volume. Notably, the mean lung dose was consistent at 93% of the prescribed dose.

The purpose of this study was to describe the successful implementation of a new IMRT based lateral‐field TBI technique at e‐SAD and to provide insights of the center's experience related to the transition and development of the technique.

## 2 MATERIALS AND METHODS

### 2.1 Beam model verification

Before commencing treatments, we had to verify whether our existing 18 MV treatment planning system (TPS) beam model, in RayStation v.10A, could accurately predict dose under e‐SSD conditions for an Elekta Synergy linear accelerator (LINAC). This involved acquiring measurements in different set‐up conditions, explained in the following Sections (2.1.1 through 2.1.3), where all measured data was compared to the TPS calculated data. This set up was reproduced by modeling the equipment in RayStation, that is, the water tank, the solid water blocks and the beam spoiler.

#### 2.1.1 Percentage depth dose and profile measurements

Prior to acquiring beam modeling data, point dose measurements were obtained with a calibrated CC13 ionization chamber in a 30 × 30 × 90 cm^3^ solid water phantom to obtain absolute dose measurements. These measurements were performed with field sizes (FS) ranging from 2 × 2 cm^2^ to 40 × 40 cm^2^ (defined at the isocenter) at depths of 3, 10, and 20 cm. The parameters for each measurement are described in Table 1.

**FIGURE 1:**
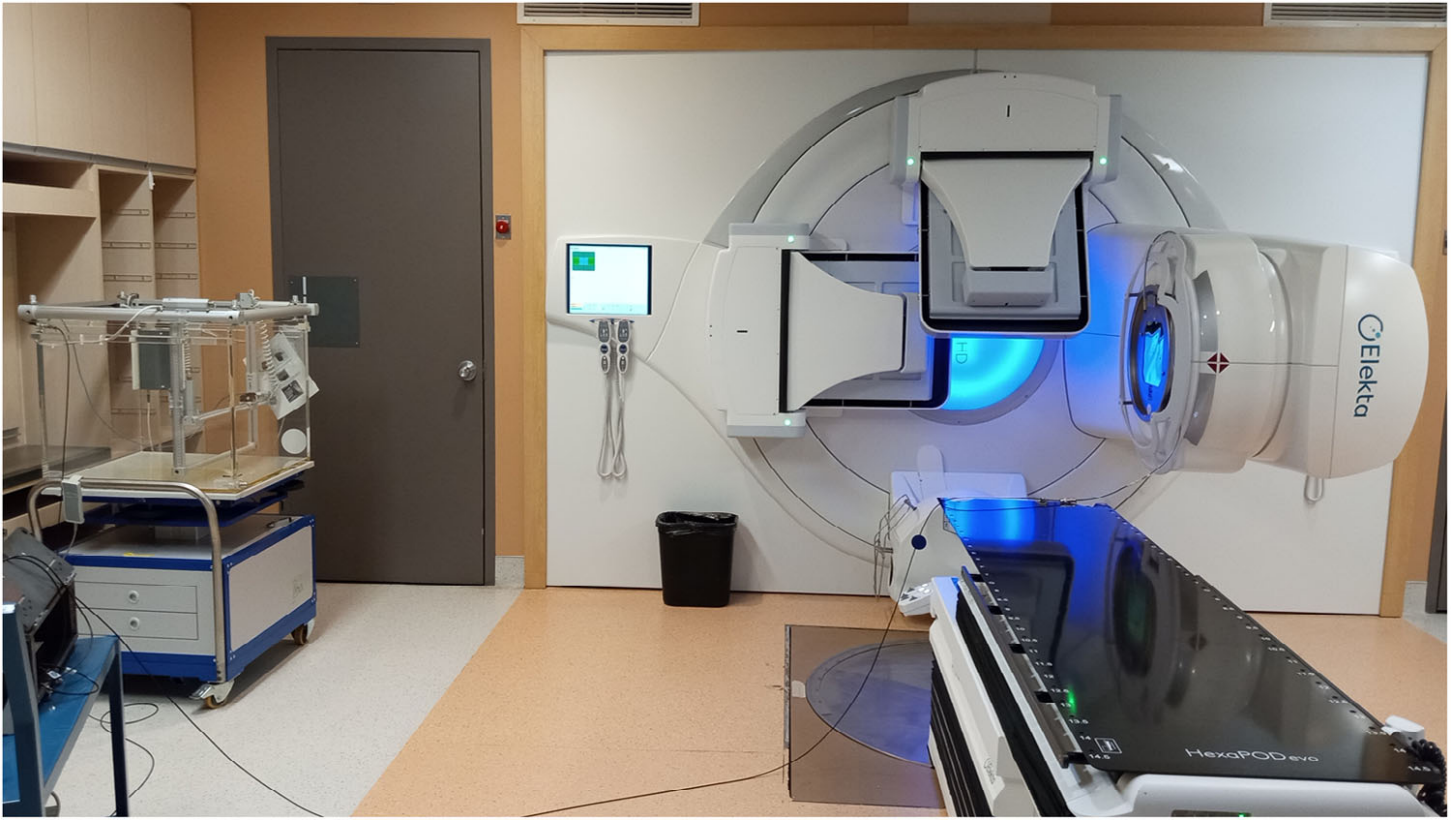
Water tank set‐up for measurements at an e‐SSD of 365 cm and a gantry angle of 270° to prepare for PDD, profile, and transmission measurements. [LINAC depicted in the image is an Elekta Versa HD as the Elekta Synergy LINAC used to treat patients has been decommissioned].

Percentage depth dose (PDD) curves, in‐plane, and cross‐plane profiles were acquired at an e‐SSD of 365 cm in a Blue Phantom 2 IBA water tank with a CC13 ionization chamber, as shown in Figure 1. This distance was chosen based on the maximum allowable in‐room distance between the LINAC and the wall. PDD water tank measurements did not include surface nor build‐up region data points. This was due to the thickness of the water tank and a mechanical constraint within the tank, which prevented the CC13 chamber from acquiring surface measurements and being positioned right at the edge of the tank. The acrylic thickness of the water tank was 1.6 cm and the distance between the edge of the tank and the point of effective measurement of the chamber was 3.6 cm. Additional measurements in solid water were required to complete the missing PDD information, discussed in Section 2.1.2.

PDD, in‐plane and, cross‐plane profiles were obtained at a series of FS described in Table 1. A 4 ft × 8 ft × 1.5 cm thick PMMA beam spoiler was positioned 10 cm anterior to the water tank, the gantry angle was set to 270° and the collimator settings varied from 0° and 45°. The purpose of the beam spoiler was to increase dose to the surface of the patient by adding a build‐up material. Transmission measurements were also acquired at various positions in the water tank to evaluate out‐of‐field dose (intra‐leaf and inter‐leaf transmission, jaw transmission and scattering).

**TABLE 1:** PDD, in‐plane, and cross‐plane profile description.

Profiles	Field size (cm^2^)	Tank position with respect to cross‐hairs	Depth (cm)
PDD, In‐plane profile, and Cross‐plane profile	2 × 2	Centered	3, 10, 20
	5 × 5	Centered	
	10 × 10	Centered	
	20 × 20	Centered	
	20 × 10	Centered	
	10 × 20	Centered	
	30 × 15	Centered	
	30 × 30	Centered	
	40 × 40^a^	Centered + off‐axis	

^a^ Additional off‐axis measurements for the 40 × 40 FS were taken by moving the water tank longitudinally in increments of 20 cm to obtain data for the entire lateral field.

#### 2.1.2 Dose‐to‐surface and build‐up measurements

**FIGURE 2:**
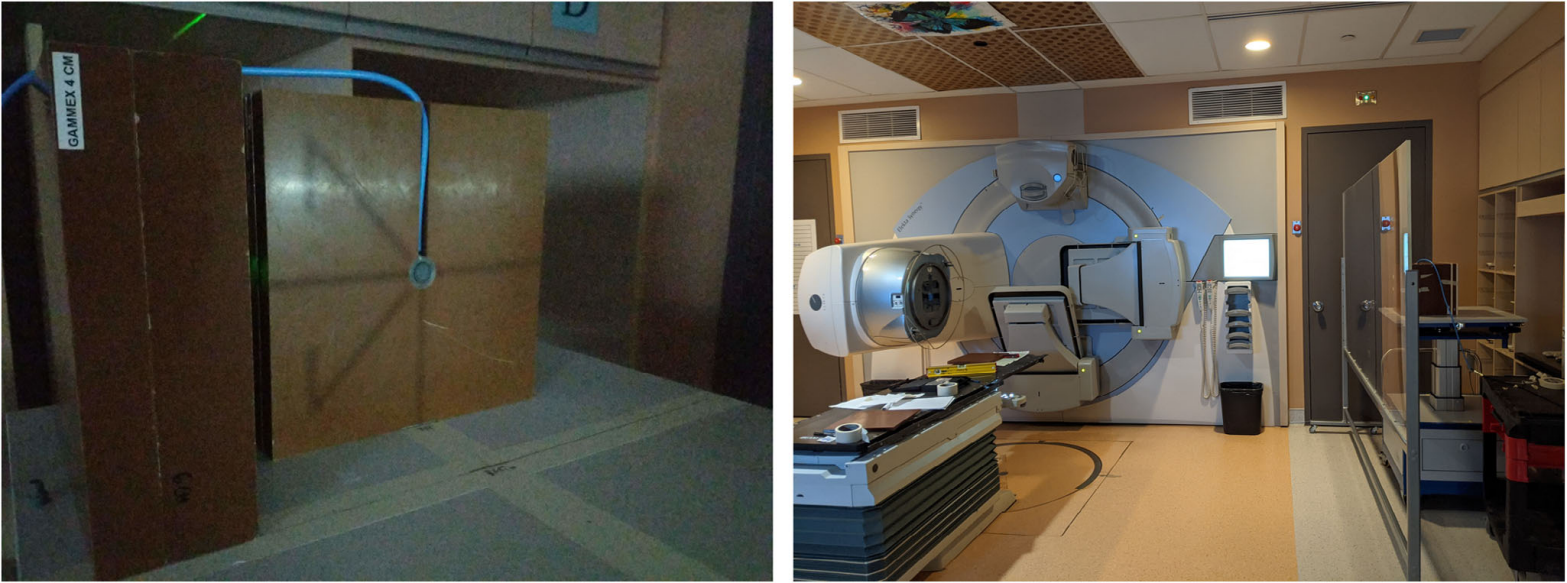
(Left) Solid water blocks with a parallel‐plate chamber centered with respect to the intersection of the cross‐hairs and (Right) set‐up for surface dose and build‐up region measurements. [LINAC depicted in the image is an Elekta Synergy].

Surface dose and build‐up region measurements were obtained independently in blocks of 30 × 30 × 30 cm^3^ solid water with an Advanced Markus parallel‐plate chamber at an e‐SSD of 365 cm and a FS of 10 × 10 cm^2^ (at isocenter). To evaluate the surface dose, the chamber was placed on the surface of the phantom, at the intersection of the projected cross‐hairs, as shown in Figure 2. The parallel‐plate chamber had no build‐up cap material covering the center of the chamber, which allowed for accurate surface dose measurement readings. The 10 × 10 cm^2^ FS extended beyond the blocks of solid water, accounting for maximal scatter contribution. Consequently, using a larger FS would have yielded the same result. To evaluate the build‐up region and maximum dose, thin slabs of solid water were placed in front of the chamber in increments of 0.5 cm to determine an approximation of the required thickness, which was then followed by 0.1 cm slabs to obtain the true maximum value.

Build‐up and dose‐to‐surface measurements were each obtained by placing the beam spoiler at various distances from the surface of the phantom, between 10 and 30 cm, to evaluate the impact as a function of distance from the phantom. An example of the set‐up is shown in Figure 2.

#### 2.1.3 Diagonal profile measurements

**FIGURE 3:**
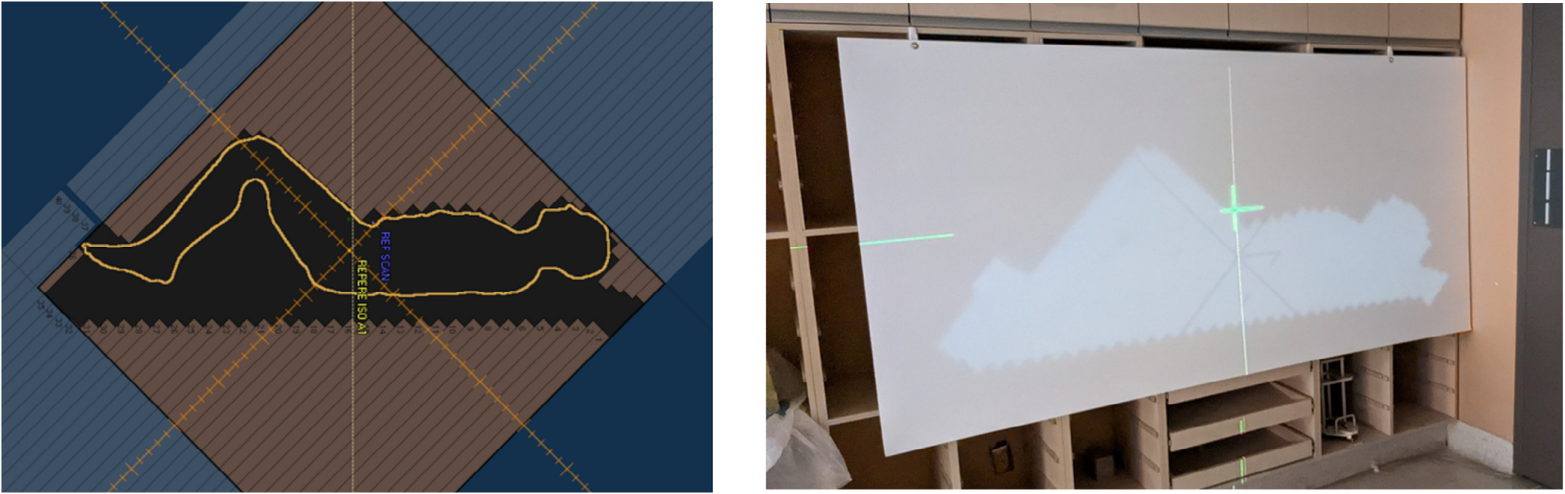
(Left) TPS BEV and (Right) light field projection of in‐room BEV for patient positioning before treatment delivery.

In order to achieve whole body coverage at an e‐SAD of 400 cm in TBI treatments, a 40 × 40 cm^2^ FS (at isocenter) was used with the collimator rotated to a 45° angle. This provided the largest possible treatment field size of 180 cm. Rotating the collimator by 45° required modeling accurate diagonal profile measurements of the field, which represented the axis of the patient during treatment, as shown in Figure 3.

Elekta Synergy LINACs did not have maximal square field sizes as the MLCs rounded the corners of the field, therefore accurate diagonal profile measurements became all the more crucial. Measurement of the diagonal profiles was obtained in a water tank under the same conditions as when the model was initially commissioned, as required by the TPS. These conditions being SSD = 90 cm with a 40 × 40 cm^2^ FS (maximal field size at isocenter) at 10 and 20 cm depth with a PTW microdiamond detector.

RayStation v.9A introduced the option to incorporate diagonal profiles, allowing for necessary adjustments and minor corrections to the existing model. Corner field data of the maximal FS at isocenter (40 × 40 cm^2^) was modified in the TPS (RayStation v.10A) mainly using the off‐axis softening and beam profile correction tools available in the RayPhysics module, to develop a more precise description of the beam profile.

### 2.2 Technique validation methods

#### 2.2.1 End‐to‐end testing

**FIGURE 4:**
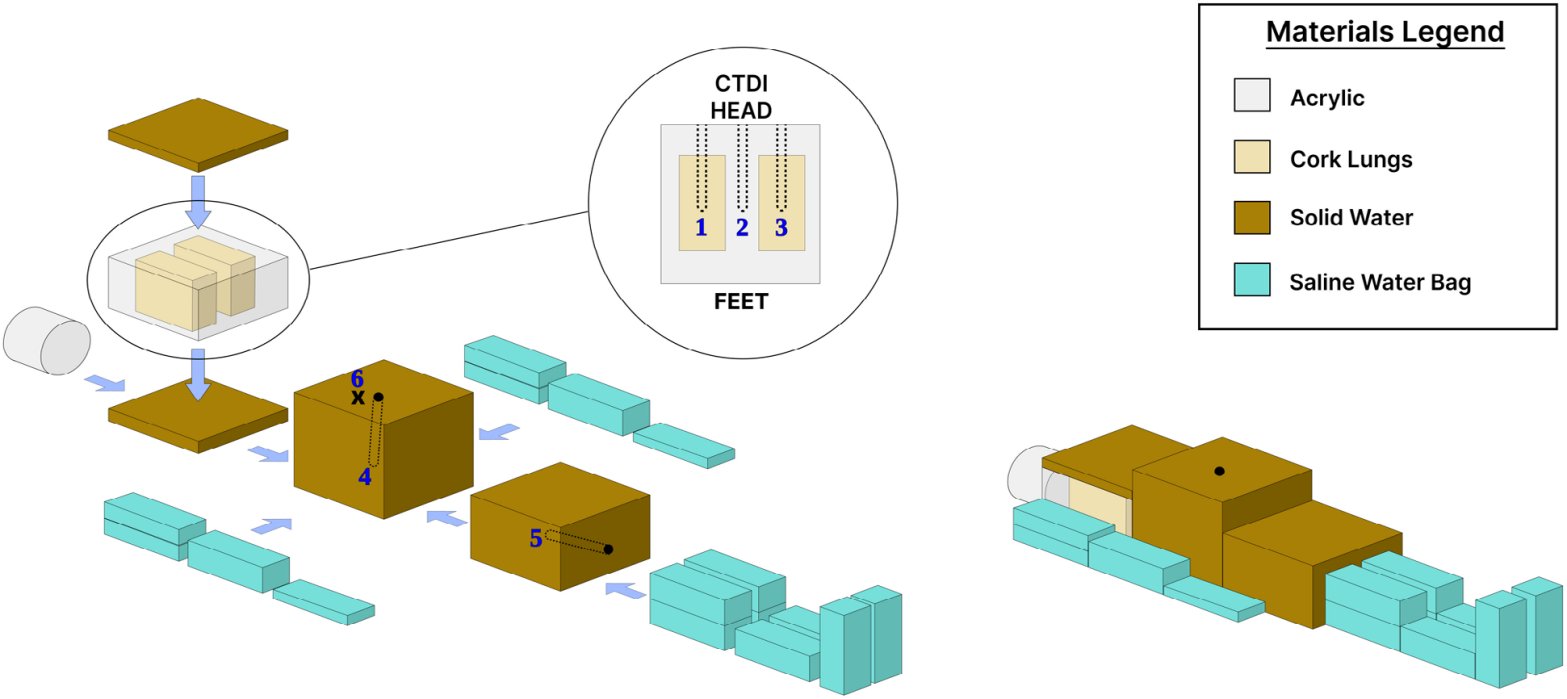
Anthropomorphic phantom made of solid water blocks, an acrylic cork block for the lungs, a CTDI phantom for the head and water saline bags to mimic the extremities of the human body. PTW Farmer chambers are identified 1 through 5 corresponding to the (1) right lung, (2), mediastinum, (3) left lung, (4) umbilical region, and (5) thigh. (6) represents the location of the diode.

End‐to‐end testing was performed prior to clinically implementing the technique. An in‐house anthropomorphic phantom was used to compare point dose measurements to calculated TPS values in different tissue‐equivalent materials with PTW N30001 farmer ionization chambers, as shown in Figure 4. The phantom was scanned and a treatment plan was created in order to evaluate the measured versus calculated data. The experimental conditions in the set‐up were identical to those employed for patient treatment, discussed in Section 2.4 (SAD = 400 cm, beam spoiler at 10 cm). Total MUs delivered were 5484 MUs, representing a prescription of 2 Gy. A Sun Nuclear ISORAD p‐type diode was used for in‐vivo measurements, positioned around the umbilical region, to ensure accurate dose comparisons prior to the deployment of the technique.

### 2.3 Treatment technique

#### 2.3.1 Simulation

**FIGURE 5:**
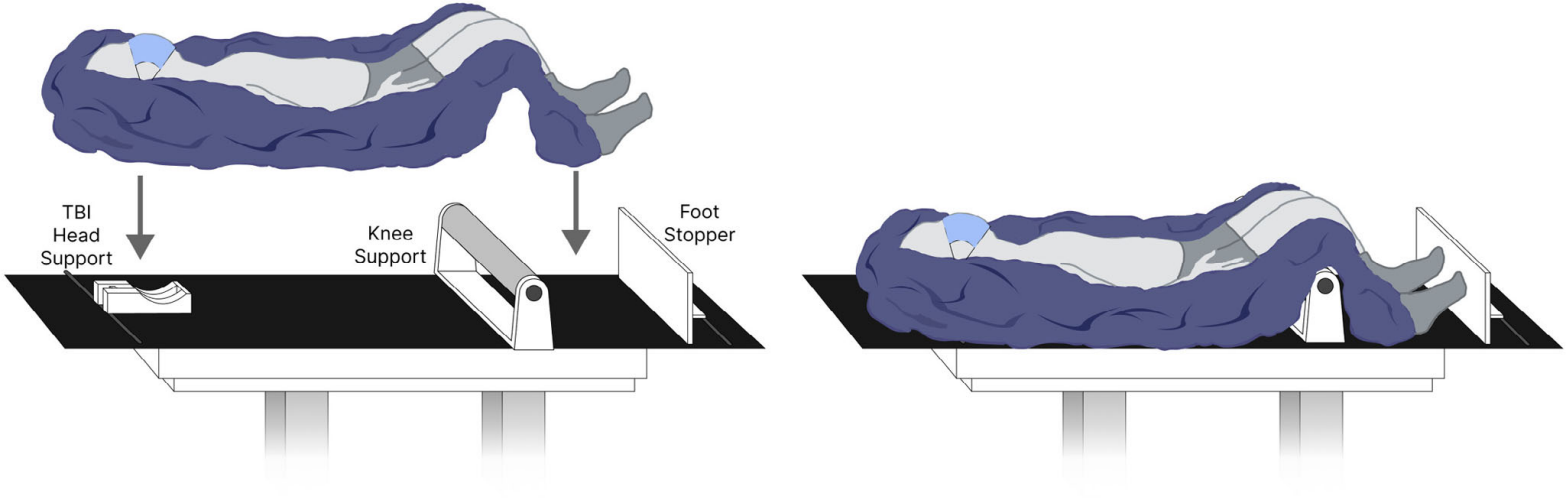
TBI patient positioning set‐up.

Patients were positioned supine in a whole body molded Vac‐Lok, as shown in Figure 5. A TBI head support was fixed on the table and used for reproducibility purposes. The knee support bent the knees at an angle which was adjustable, allowing the patient to fit in the 180 cm maximal treatment FOV at e‐SSD and to fit in the Philips Brilliance Big Bore CT simulator. If the knee bend extended outside the FOV of the CT scan, the knees might have needed to be reconstructed in the TPS and overwritten with the appropriate density. Arms were to the side of the patient for comfort and reproducibility. They were embedded in the molded Vac‐Lok to minimize uncertainty in positioning. A foot stopper was placed to ensure toes were fully irradiated and for reproducibility purposes. A marker was drawn on the Vac‐Lok to indicate the midline of the patient to help guide with in‐room positioning.

The Brilliance Big Bore CT simulator had a maximal scan length of 135 cm, therefore two separate CT scans, head‐first and feet‐first, were merged with in‐house scripting to generate a whole‐body 5 mm slice thickness scan for treatment planning. A detachable in‐house central pivoting table was designed to facilitate the acquisition of both CT scans without repositioning the patient. A head‐first CT scan was acquired with the patient in a supine position, followed by a feet‐first supine scan, which was acquired by rotating the central pivoting table by 180°.

#### 2.3.2 Treatment planning

The treatment technique was planned with an Elekta Synergy LINAC at SAD = 400 cm with the highest available energy, 18 MV. It was comprised of two open fields with a 4 cm MLC flash surrounding the patient's body in air and additional inverse planned IMRT segments to complete the treatment and shield the lungs, kidneys and brain. The number of segments for optimal plan quality was tested and 12−16 IMRT segments were chosen as an acceptable compromise between minimizing treatment time and maintaining plan quality. To increase surface dose, the 1.5 cm thick beam spoiler was placed 10 cm from the couch. The spoiler was modeled in the TPS as an accessory during the dosimetry planning process since it could not be scanned. It was given a material override of PMMA available in RayStation, with a density of 1.19 g/cm^3^.

Due to the mechanical limitations of the couch height and the desire for the cross‐hairs to be at the midpoint of the patient, the gantry was angled to 87°. A collimator angle of 135° was used to reach the maximum FOV. To minimize reproducibility errors and for practical reasons, the lateral fields were delivered on the same side of the treatment room, with the same gantry angulation. The first lateral field was delivered with the beam entering the left side of the patient and the second field, with the beam entering the right side of the patient. A geometric transformation of the treatment fields was required before transferring TPS clinical plans to our record and verify system (MOSAIQ). This was done by rotating the collimator 180° and keeping the gantry rotation angle fixed for the second treatment field.

Fractionation schemes of 12 Gy in 6 fractions twice a day, 4 Gy in 2 fractions and 2 Gy in 1 fraction daily were used depending on diagnosis, graft type and chemotherapy regimen among other determining factors. Our dosimetry criteria were based on the publication by Fog et al., which uses 12 Gy in 6 fractions.^8^ In our clinic, plans were optimized using 12 Gy in 6 fractions and scaled down to the desired number of fractions. Table 2 shows our clinical goals used for a 12 Gy in 6‐fraction treatment. The PTV is defined as the body and excludes the lungs, while the modPTV is delineated as the PTV contracted inward from the skin by a 5 mm margin.

**TABLE 2:** Ideal and acceptable clinical goals in our clinic.

	Criteria	Ideal	Acceptable
**Body**	Max D0.1cc	<14.40 Gy	15.60 Gy
	Min V9.6 Gy	>99.50%	–
**modPTV**	Min V10.4 Gy	>95.00%	95.00%
	Min V10.9 Gy	>95.00%	–
	Max 12.8 Gy	<10.00%	15.00%
**Lungs**	Max V12.0 Gy	<2.00%	–
	Min V10.2 Gy	>95.00%	–
	Mean	<11.16 Gy	–
**Brain**	Max D0.1cc	<12.36 Gy	12.60 Gy
	Mean	<11.40 Gy	11.80 Gy
	Max V12Gy	<5.00%	ALARA
**Kidneys**	Max D0.1cc	<12.36 Gy	12.60 Gy
	Mean	<11.40 Gy	11.80 Gy
	Max V12Gy	<5.00%	ALARA

#### 2.3.3 Pre‐treatment plan checks

Pre‐clinical QA was performed prior to every patient treatment. It consisted of comparing point dose measurements with the TPS in a given set‐up.

A calibrated Farmer and a 30 × 30 × 30 cm^3^ solid water phantom were placed under treatment conditions (SAD = 400 cm, FS = 40 × 40 cm^2^, depth = center of the phantom). The phantom was moved longitudinally to the appropriate position on the treatment couch, determined in the TPS to avoid any gradient regions and ensuring that we verified that the geometrical field transformation was taken into account. The treatment was delivered and the measured values were compared with the calculated values with a 3% passing threshold, per lateral field.

### 2.4 Treatment delivery

Beam‐on time remained on average 39 min with a minimum treatment time of 35 min and a maximum of 45 min. Total MUs were kept under 6000. The LINAC dose‐rate was set to 140 MU/min in MOSAIQ to achieve an instant dose‐rate of 8.75 cGy/min at 400 cm, similar to the dose rate obtained with our previous Co‐60 unit treatments (14.20 cGy/min in 2016 to 6.30 cGy/min in 2022).

#### 2.4.1 In‐room patient positioning

A vertical mounted laser on the ceiling was used to align the center of the patient at 400 cm SAD, using the lines drawn on the Vac‐Lok as a reference of patient midline.

Pre‐treatment imaging with cone‐beam CT or MV portal imaging prior to treatment delivery was not possible. For this reason, light field projections with the MLCs (using a beam's eye view (BEV)) in their initial position were used for in‐room patient positioning for both lateral fields, as shown in Figure 3. Millimetric positioning accuracy was not possible, therefore the robustness was consequently evaluated using a tool in the TPS by applying 1 cm shifts from the isocenter in all possible directions (laterally, longitudinally, and vertically) and evaluating the dosimetric impact on organs at risk (OAR), coverage and homogeneity of the plan. Data analysis beyond 1 cm shifts was performed but not reported due to the unrealistic nature of set‐up inaccuracies exceeding 1 cm.

The beam spoiler was placed 10 cm from the couch for an average sized patient and 15 cm for larger patients, as the Vac‐Lok extended beyond the couch. The spoiler had an associated bar code, which acted as an interlock prior to starting treatment.

#### 2.4.2 In‐vivo dosimetry

In‐vivo dosimetry was performed for every patient using a Sun Nuclear ISORAD p‐type diode, which was placed mid‐plane on the patient's abdomen. A diode was modeled in the TPS and measured dose was compared to the calculated value with a 5% passing threshold. The diode reading was corrected for temperature variations but not for obliquity effects. As a result, temperature was measured at the end of delivery of each incidence. Due to the cylindrical design of the diode and the calibration performed in the same geometry as for treatment, there was no need for angular corrections.

## 3 RESULTS AND DISCUSSION

### 3.1 Beam model verification

Our existing 18 MV model, under standard conditions, successfully calculated dose that matched measurements under e‐SSD conditions. These calculations were validated based on the measurements performed and are discussed in Sections 3.1 and 3.2.

#### 3.1.1 PDD and profile measurements

Absolute point dose measurement results were reported as a function of depth for all FS and compared with TPS values. Results are shown in Table A1 in the Appendix. The most important FS for beam model verification was the 40 × 40 cm^2^ FS, which had point dose percent differences under 2.2% for all three depths.

**FIGURE 6:**
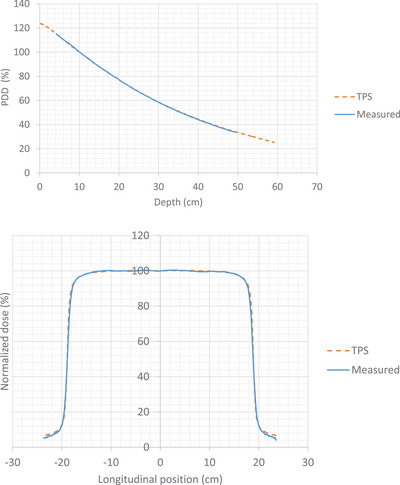
PDD (top) and in‐plane (bottom) measurements between the TPS (orange dash) and measured data (blue solid) for a 10 × 10 cm^2^ FS at isocenter at an e‐SSD of 365 cm at 3 cm depth. The PDD measurements are normalized at 10 cm depth and the in‐plane profile measurements are normalized on the central axis. PDD measurements did not include the build‐up region.

The CC13 PDD and profile measurements agreed with less than a 2% difference for all FS measurements, including off‐axis measurements, when comparing to the standard TPS model. Note that off‐axis data were collected for one side of the field, assuming the field was symmetrical. Results are shown in Figure 6 for a 10 × 10 cm^2^ FS defined at the isocenter, at 3 cm depth at an e‐SSD of 365 cm.

#### 3.1.2 Dose‐to‐surface, build‐up measurements, and optimal beam spoiler position

**FIGURE 7:**
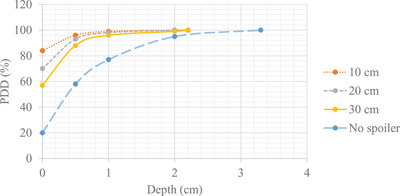
Surface‐dose and build‐up region to missing water tank PDD data, for differing beam spoiler distances from the solid water phantom—orange (10 cm), gray (20 cm), yellow (30 cm), and blue (no spoiler).

Table 3 and Figure 7 show the PDD results of dose‐to‐surface at e‐SSD obtained by moving the beam spoiler at different positions from the solid water phantom. The closer the spoiler to the phantom, the greater the surface dose. The closest possible distance between the spoiler and treatment couch was 10 cm. This distance was found to be the most optimal, with highest dose‐to‐surface and shallower maximum dose, $d_{max}$ (quicker build‐up).

**TABLE 3:** Surface‐dose and 5 mm build‐up measurements for different beam spoiler positions and calculated percent difference between TPS and measured PDD values.

Dose‐to‐surface		
Position of beam spoiler from water phantom (cm)	PDD (%)	Difference between measured and TPS (%)
No spoiler	19.8	−22.5
10	83.8	−8.7
20	69.5	−16.8
30	57.2	−20.0

Dose with 5 mm build‐up		
Position of beam spoiler from water phantom (cm)	PDD (%)	Difference between measured and TPS (%)
No spoiler	57.9	−0.7
10	95.5	−1.0
20	93.3	1.2
30	87.9	4.0

*Note* that the difference between the measured and TPS values was calculated as follows: (Measured—TPS)/TPS.

The measured surface dose, at the front surface of the collecting volume, was compared to the TPS calculated dose, at the surface. The smallest percent difference was found with the spoiler positioned 10 cm away. However, these results should be interpreted with caution due to the inaccuracy of the TPS model for surface dose calculations. As the beam spoiler distance increased, the larger the surface dose discrepancy between RayStation and measured values. The TPS over predicted dose to the surface at all spoiler locations. However, as shown in Table 3, starting from a 5 mm build‐up of solid water, percent difference was less than 4% between TPS and measured data.

#### 3.1.3 Diagonal measurements

**FIGURE 8:**
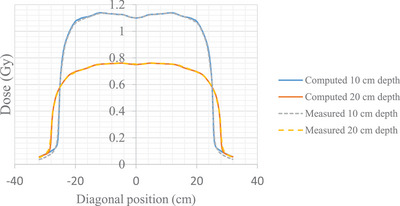
Diagonal profiles for a 40 × 40 cm^2^ FS at isocenter at 10 and 20 cm depth for measured data (gray and yellow dash) and TPS computed after adjustments (blue and orange solid).

Through data acquisition, our beam model was thoroughly verified and modified to accurately characterize the corner edges for a 40 × 40 cm^2^ FS as described in Section 2.1.3. Figure 8 shows the measured data and TPS computed data after the necessary adjustments.

### 3.2 Technique validation methods

#### 3.2.1 End‐to‐end testing

The corresponding measurements of the set‐up in Figure 4, Section 2.2.1 are summarized in Table 4. These dose values were measured for a total treatment delivery. The largest percent difference in any simulated organ material with a Farmer chamber of measured versus TPS data was 2.1%.

**TABLE 4:** The percent difference between the measured and calculated dose with respect to the different chamber positions as shown in Figure 4, Section 2.2.1.

Material	Difference between measured and calculated dose (%)
(1) Umbilical	1.4
(2) Thigh	1.3
(3) Right lung	1.7
(4) Left lung	1.2
(5) Mediastinum	2.1

#### 3.2.2 Pre‐clinical QA

Pre‐clinical QA is essential when integrating a new technique, particularly for TBI, as large doses are administered to the entire body. Mean point dose measurements versus TPS calculated for 17 patients were found to be ‐0.74 ± 1.62% for the left lateral field and ‐0.26 ± 2.03% for the right lateral field, with our institutional tolerance level at 3% per lateral field.

### 3.3 Clinical results

#### 3.3.1 Treatment delivery

Total MU delivery was on average 4807 MUs, for 17 patients treated to date. The average clinical goal results are shown in Table 5. For patients who did not receive the standard 12 Gy in 6 fractions, their clinical results were scaled for inclusion in Table 5.

**TABLE 5:** The average clinical results with standard deviation (SD) obtained for 17 patients.

	Criteria	Average clinical goals with SD	Ideal clinical goals
**Body**	Max D0.1cc	14.25 ± 0.20 Gy	<14.40 Gy
	Min V9.6 Gy	99.90 ± 0.03%	>99.50%
**modPTV**	Min V10.4 Gy	99.80 ± 0.11%	>95.00%
	Min V10.9 Gy	97.21 ± 0.50%	>95.00%
	Max V12.8 Gy	4.03 ± 1.77%	<10.00%
**Lungs**	Max V12.0 Gy	0.60 ± 0.45%	<2.00%
	Min V10.2 Gy	99.92 ± 0.23%	>95.00%
	Mean	11.08 ± 0.08 Gy	<11.16 Gy
**Brain**	Max D0.1cc	11.82 ± 0.15 Gy	<12.36 Gy
	Mean	11.16 ± 0.05 Gy	<11.40 Gy
	Max V12Gy	0.01 ± 0.02%	<5.00%
**Kidneys**	Max D0.1cc	11.99 ± 0.14 Gy	<12.36 Gy
	Mean	11.31 ± 0.04 Gy	<11.40 Gy
	Max V12Gy	0.32 ± 0.62%	<5.00%

Open field delivery constituted approximately 60% of the total dose. To shield the lungs and kidneys, and reduce dose to the brain, IMRT segments were used to administer the remaining 40%. As shown in Table 2, we accepted 93% of the prescribed dose as mean lung dose with less than 2% of the lung volume receiving 12 Gy. The maximum dose to the brain and kidneys was kept as low as reasonably achievable (ALARA) with a mean dose less than 95% of the prescribed dose. These clinical values were based on the study by Fog et al. and on our local experience.^8^ For our institutional Co‐60 TBI treatments, dose was prescribed to the midpoint of the patient, that is, the umbilical region. Wood's metal (Cerrobend) was used to shield the lungs, where the 1 cm thick cutouts covered the lung area minus 1 cm from the edge of the ribs, diaphragm, and clavicles, therefore ideally blocking up to 25% of the prescribed dose at the midpoint of the lung in an average sized patient. This corresponded to approximately 75% of the prescribed dose to the center of the lung and between 90% and 110% on the periphery of the lung with a mean dose of approximately 80% for the entirety of the lung. For much larger patients, the mean dose to the lung value easily increased to 95% of the prescribed dose. These values were estimates calculated through heterogeneous phantoms at the time of clinical implementation. Historically, in the literature, for a 12 Gy in 6 fraction regimen, the mean dose to the lung was reduced to 8–9 Gy to decrease risk of interstitial pneumonitis.^12^ For the IMRT lateral‐field technique, efforts were made to reduce the mean lung dose to levels more commonly reported in literature. However, given the choice of the technique, reducing the mean lung dose below 11 Gy often resulted in a loss of PTV coverage of less than 10.4 Gy. To maintain adequate coverage of the PTV, we established a minimum lung dose of 10.2 Gy (equivalent to 85% of the prescription dose) with a mean dose of 11.16 Gy. This approach aligned with our previous technique's outcomes and the data reported by the Fog et al. group.^8^

All 17 patients, with the exception of one, met the ideal clinical goals stated in Table 2. The treatment plan for the first treated patient was unable to meet the optimal maximum dose constraint value to the body (less than 14.40 Gy), yet it remained within our acceptable limit (15.60 Gy). This was mainly due to the lack of experience when creating treatment plans for larger patients in this particular set‐up. Incorporating additional IMRT segments (from 12 to 16) and adjusting the objective weights led to an improved inverse optimization solution for these cases.

#### 3.3.2 In‐room patient positioning: Robustness evaluation

Table 6 provides a summary for all patients treated to date for each clinical goal, showing the range of values obtained when applying 1 cm shifts in each axis. Based on the results, an error of 1 cm was found to be within our clinically acceptable tolerance on target coverage and OAR dose. These results met the standards for treatment by the radiation oncology medical team.

**TABLE 6:** Range obtained for each clinical goal when applying 1 cm shifts from the isocenter.

	Criteria	Range	Acceptable clinical goals
**Body**	Max D0.1cc	14.55−15.68 Gy	15.60 Gy
	Min V9.6 Gy	99.82−99.92%	–
**modPTV**	Min V10.4 Gy	98.84−99.80%	95.00%
	Min V10.9 Gy	92.50−95.90%	–
	Max V12.8 Gy	4.15−9.73%	15%
**Lungs**	Max V12.0 Gy	0.02−3.80%	–
	Min V10.2 Gy	88.45−100.00%	–
	Mean	10.96−11.34 Gy	–
**Brain**	Max D0.1cc	11.61−12.1 Gy	12.60 Gy
	Mean	11.09−11.26 Gy	11.80 Gy
	Max V12Gy	0.00%−0.16%	ALARA
**Kidneys**	Max D0.1cc	12.15−12.93 Gy	12.60 Gy
	Mean	11.29−11.78 Gy	11.80 Gy
	Max V12Gy	0.42%−21.99%	ALARA

The dosimetric impact of a systematic error remained within the acceptable limit for each patient. This implied that patients treated with random error in patient positioning within these tested limits would exhibit only minor effects on the dosimetric outcome of their treatment plan. Based on our previous treatment technique and the correlation with patient outcomes, the analysis was centered around the V12Gy and mean dose values. The V12Gy to the kidneys experienced significant variability but was still considered permissible, specifically in comparison to our Co‐60 technique where no kidney shielding was possible. The V12Gy to the lung remained below 4%, which was clinically acceptable and validated by the medical team. As for the mean dose values, the kidneys remained below the acceptable value of 11.80 Gy in all cases and below 95% of the prescribed dose for the lungs. These values were consistent with our previous method, and therefore clinically accepted.

The robustness evaluation demonstrated that a lateral shift in patient positioning would have minimal impact on the dosimetry. In 12% of cases, a longitudinal 1 cm shift had the greatest influence, while in 88% of cases, the most impactful shift was found to be along the vertical axis (i.e., couch height) in terms of coverage, OARs and hot spots. More specifically, the greatest discrepancies were found when moving the couch in the upward direction by 1 cm.

#### 3.3.3 In‐vivo dosimetry

In‐vivo dosimetry was performed for every patient. The diode readings over‐predicted dose in all 17 cases. After investigating the situation, the greater disagreement between diode measurement and TPS prediction was due to a temperature dependence of 0.5%/°C. To date, with these corrections in place, 35 additional patients have been treated. These results yielded a percent difference of 1.55 ± 3.43% for the left lateral field and 1.75 ± 3.43% for right lateral field, which was within the 5% institutional threshold.

### 3.4 Patients treated to date

#### 3.4.1 Patient characteristics

A total of 17 patients with an average age of 43 were treated between June and December 2022. Out of the 17 patients, 53% were male and 47% were female. The average body mass index (BMI) was 25.

The diagnosis along with corresponding total dose are included in Table 7. The selected TBI dose for each patient was prescribed according to the conditioning protocol, which was selected by our stem cell transplant team depending on patient characteristics, diagnosis and type of bone marrow transplant.

**TABLE 7:** Diagnosis and total dose administered at 2 Gy per fraction for the 17 affected patients in our clinic.

Diagnosis	Number of patients	Total dose (Gy)
ALL	5	12
	1	4
AML	2	12
	1	4
MDS	1	2
MM	3	2
APLASIA	1	2
T NHL	1	12
	1	4
	1	2

Abbreviations: ALL, acute lymphoid leukemia; AML, acute myeloid leukemia; MDS, myelodysplastic syndrome; MM, multiple myeloma; T NHL, T Non Hodgkin Lymphoma.

#### 3.4.2 Patient outcomes

To date, TBI treatments using the IMRT based lateral‐field e‐SAD technique are well tolerated without any unexpected acute side effects in comparing to the previous Co‐60 treatment method.

## 4 CONCLUSION

An IMRT based lateral‐field e‐SAD technique was developed in our clinic for patients undergoing TBI. This technique was inspired by the Fog et al. experience and has demonstrated enhancements in comparison to our previous Co‐60 TBI method in terms of treatment planning and radiation safety protocols.^8^

Patient treatment planning was made possible through RayStation following rigorous beam model validation testing methods. The commissioning process of the new IMRT‐based lateral field e‐SAD technique for TBI was successful with less than a 2% difference between measured and calculated PDD and profile data. These measurements resulted in accurate treatment plans and delivery with in‐vivo diode measurements of less than a 5% difference.

With the use of a TPS, we had the ability to create personalized treatment plans with a better visualization and analysis of the plan administered to the patient and therefore, improved quality of the delivered plan compared to Co‐60. Our IMRT delivery technique no longer required the use of Wood's metal, as shielding was done through segments and had shown improvement with dose homogeneity in comparing to the previous Co‐60 technique. This was mainly due to the controlled beam modulation, e‐SSD and to the available use of a higher beam energy.

The clinical implementation involved a multidisciplinary and collaborative approach, emphasizing frequent communication among radiation therapists, dosimetrists, physicists and physicians. Based on our experience, the information presented in this article can function as a point of reference for other institutions aiming to introduce TBI within their clinical setting.

## AUTHOR CONTRIBUTIONS

This project aims to reflect the contribution and implication of a large multidisciplinary team of radiation therapists, dosimetrists, physicists, and physicians to develop and implement an IMRT lateral‐field e‐SAD TBI technique at our center. Anastasia Kolokotronis (Physicist) wrote the manuscript and contributed with Malik Brunet‐Benkhoucha and Étienne Roussin (Physicists) to the data collection, data analysis, and clinical workflow of the technique. Eve‐Lyne Marchand and Maryse Bernard (Radiation Oncologists) were this project's medical lead in selecting the IMRT lateral‐field e‐SAD technique that was developed and contributed to the patient data analysis and determining dosimetry clinical objectives. Julie St‐Pierre (Dosimetrist) contributed to the treatment and dosimetry planning aspects. All authors read, reviewed, and approved the manuscript.

## CONFLICT OF INTEREST STATEMENT

The authors declare no conflicts of interest.

## APPENDIX 1

### Absolute point dose measurement results reported as a function of depth for all fs spanning 2 × 2 to 40 × 40 (at isocenter) and compared with tps values

**TABLE A1:** Percent difference of the absolute point dose measurements compared to TPS predicted values.

Field size (cm^2^)	Depth of chamber (cm)	Difference between measured and TPS (%)
2 × 2	3	1.9
	10	0.1
	20	0.0
5 × 5	3	3.3
	10	0.9
	20	0.3
10 × 10	3	1.3
	10	0.1
	20	−0.5
20 × 20	3	2.9
	10	1.9
	20	1.0
30 × 15	3	3.0
	10	1.7
	20	1.2
40 × 15	3	3.2
	10	2.2
	20	1.4
30 × 30	3	1.0
	10	−0.3
	20	−0.7
40 × 40	3	2.2
	10	1.3
	20	0.6
